# Association Between Subclinical Hypothyroidism and MASLD: A Systematic Review and Meta-Analysis

**DOI:** 10.1155/ijh/8133686

**Published:** 2025-11-29

**Authors:** Gedion Yilma Amdetsion, Chun-Wei Pan, Hiwot Tebeje, Abhin Sapkota, Shreyas Nandyal, Vikram Kotwal

**Affiliations:** ^1^Department of Medicine, John H Stroger Jr Hospital of Cook County, Chicago, Illinois, USA; ^2^Division of Gastroenterology and Hepatology, Department of Medicine, University of Massachusetts Chan Medical School, Worcester, Massachusetts, USA; ^3^Department of Epidemiology and Biostatistics, Washington University in St. Louis, St. Louis, Missouri, USA; ^4^Division of Gastroenterology and Hepatology, Department of Medicine, John H Stroger Jr Hospital of Cook County, Chicago, Illinois, USA

**Keywords:** liver steatosis, meta-analysis, metabolic dysfunction associated liver disease, subclinical hypothyroidism

## Abstract

**Background:**

Metabolic dysfunction–associated steatotic liver disease (MASLD) is a highly prevalent chronic liver disorder that can progress to cirrhosis and hepatocellular carcinoma. Although overt hypothyroidism has been identified as a MASLD risk factor, the impact of subclinical hypothyroidism (SCH), which affects approximately 4.6% of US adults, remains unclear. We conducted a systematic review and meta-analysis to determine whether SCH is independently associated with MASLD and to inform targeted screening recommendations.

**Methods:**

We systematically searched PubMed, Embase, Cochrane, ClinicalTrials.gov, and Web of Science up to June 2025 for studies evaluating the association between SCH and MASLD. Random-effects meta-analyses were performed on eligible cross-sectional and longitudinal studies.

**Results:**

We screened 537 records and ultimately included 10 high-quality studies with 71,332 participants. Overall, 22.3% had MASLD and 7.7% had SCH. In cross-sectional analyses (*n* = 39,814), SCH was linked to 46% higher odds of MASLD (pooled OR = 1.46, 95% CI 1.23–1.73; *I*^2^ = 36%). Across four longitudinal cohorts (*n* = 31,518), SCH raised the risk of incident MASLD by 59% (pooled HR = 1.59, 95% CI 1.05–2.40). Limiting the analysis to prospective studies strengthened the association (HR = 1.90, 95% CI 1.50–2.39) and eliminated heterogeneity (*I*^2^ = 0).

**Conclusions:**

This meta-analysis provides evidence that SCH is associated with both higher odds of prevalent MASLD and increased risk of incident MASLD. Clinicians should consider routine screening for MASLD in patients with SCH and vice versa.

## 1. Introduction

Affecting roughly 25%–30% of the global adult population, metabolic dysfunction-associated steatotic liver disease (MASLD) is one of the leading causes of cirrhosis [1]. Defined by hepatic steatosis in individuals with one or more metabolic risk factors such as obesity, Type 2 diabetes, and dyslipidemia after excluding significant alcohol use and other chronic liver diseases, MASLD arises from multifactorial mechanisms including insulin resistance, lipotoxicity, chronic low-grade inflammation, oxidative stress, genetic and epigenetic influences, and lifestyle factors [2]. Given its high prevalence and the fact that hepatic fibrosis advances in roughly 36% of MASLD patients, a systematic identification and management of all relevant risk factors is essential for early detection and halting disease progression [3]. Among these, hypothyroidism has emerged as a significant potential risk factor [4].

A meta-analysis by Mantovani et al. that included patients with both overt and subclinical hypothyroidism (SCH) found a 39% higher incidence of MASLD [4]. The relationship between hypothyroidism in general and MASLD was initially attributed to shared pathophysiological factors such as insulin resistance and dyslipidemia. However, analyses by Mantovani et al. and Wang et al. revealed that this association remains significant even after adjusting for diabetes and dyslipidemia, suggesting that additional mechanisms may be at play [4, 5]. For instance, thyroid hormone deficiency can alter hepatic lipid metabolism by reducing genes involved in mitochondrial fatty acid *β*-oxidation, decreasing hepatic lipoprotein lipase activity, and impairing the biliary excretion of cholesterol [6, 7]. Moreover, chronic low-grade inflammation and shifts in adipokine profiles have been implicated in the pathogenesis of both hypothyroidism and MASLD, further underscoring the complexity of this relationship [4, 8].

Although overt hypothyroidism has been clearly linked to MASLD, the association with SCH remains inconclusive. SCH affects approximately 4.6% of adults in the United States making it far more common than overt hypothyroidism [9]. Moreover, whereas patients with overt hypothyroidism often attain a euthyroid state through hormone replacement, individuals with SCH typically exhibit persistently elevated TSH [10, 11]. However, the relationship between SCH and MASLD remains incompletely understood due to inconsistencies in study findings, heterogeneity in study design, small sample sizes, and a lack of longitudinal data [12]. These limitations underscore the need for a comprehensive meta-analysis to clarify the association between SCH and MASLD. By pooling data from multiple studies, a meta-analysis can enhance statistical power, address heterogeneity, improve generalizability, and resolve conflicting results, thereby providing a clearer consensus on the relationship between these two conditions [13]. This meta-analysis is aimed at synthesizing existing evidence to better understand the interplay between SCH and MASLD, ultimately informing clinical practice and guiding future research.

## 2. Methods

The protocol of this systematic review was registered on Prospero at CRD420251116591.

### 2.1. Data Sources and Searches

This systematic review has been performed following the Preferred Reporting Items for Systematic Reviews and Meta-Analyses (PRISMA) and the Meta-analysis Of Observational Studies in Epidemiology guidelines [14, 15]. We systematically searched PubMed, Embase, Cochrane, clinicaltrials.gov, and Web of Science from database inception to June 2025. Keywords used in the literature search included combinations of terms related to “Subclinical hypothyroidism” and “metabolic dysfunction-associated steatotic liver disease” to identify relevant studies. Searches were restricted to human studies. No language restrictions were imposed. For the full search strategy with complete search terms and syntax for each database, please refer to Supporting Information 2: Table S1. Furthermore, we reviewed references from relevant review articles to identify additional eligible studies not covered by the original database searches. Because this meta-analysis used only previously published, anonymized data, formal ethics approval and informed consent were not required.

### 2.2. Study Selection

Eligible studies were included in the meta-analysis if they met the following inclusion criteria: (1) prospective/retrospective cohort studies or cross-sectional studies; (2) studies that reported ORs or HRs with 95% CI values for the outcome of interest; (3) the diagnosis of MASLD was based on liver biopsy, imaging techniques or International Classification of Diseases (ICD) codes; and (4) the diagnosis of SCH was based on TSH levels above 4 and normal free T4 levels. Subjects included in the meta-analysis were of either sex without any race or ethnicity restrictions.

The exclusion criteria of the meta-analysis were as follows: (1) case reports, reviews, practice guidelines, commentaries, or editorials; (2) studies that enrolled patients under the age of 18; and (3) no clear definition of SCH.

We followed the PRISMA guidelines to identify studies assessing the relationship between SCH and MASLD. Two authors (G.A and C.P) independently reviewed the titles and abstracts of the studies identified in the primary search and excluded those that did not address the research question, based on prespecified exclusion and inclusion criteria. The full text of the remaining articles was reviewed to determine whether they contained relevant information. Any discrepancy in article selection was resolved by consensus and in discussion with a third co-author (H.T). The bibliographic sections of the selected articles, as well as systematic and narrative articles on the topic, were searched for additional relevant articles.

### 2.3. Data Extraction and Quality Assessment

Data from studies eligible for the aggregate data meta-analysis were independently extracted by two investigators (G.A. and C.P.). Any disagreements between investigators about the inclusion of eligible studies were resolved by consensus and a third author (V.K.) if needed.

For each eligible study, we extracted data on publication year, sample size, country, mean duration of follow-up, mean age, mean BMI, biological sex, mean TSH, how the MASLD diagnosis was made, the number of patients with MASLD, TSH cut-off used to diagnose SCH, the number of patients with SCH, the hazard ratio/risk ratio, and the covariates that were accounted for. In the case of multiple publications of the same database, we included the most up-to-date or comprehensive information.

The overall quality of the included non-randomized studies was assessed using the Newcastle–Ottawa Scale (NOS). Two independent authors evaluated each study, and any scoring discrepancies were resolved through discussion and consensus. The NOS is a validated tool for appraising observational research, assigning up to nine stars across three primary domains: selection, comparability, and outcome (or exposure, depending on the study design). A higher star rating generally indicates a lower risk of bias and higher methodological quality [16, 17].

### 2.4. Data Synthesis and Analysis

The primary outcome measure of the meta-analysis was to assess if SCH is associated with MASLD. The adjusted OR/HRs of all eligible studies were pooled, and an overall ES estimate was calculated using a random-effects model when able. The statistical heterogeneity among studies was evaluated by the *χ*^2^ test and the *I*^2^ statistic, which estimates the percentage of variability across studies due to heterogeneity rather than chance alone. The proportion of heterogeneity accounted for by between-study variability was assessed using the *I*^2^ statistic and adjudicated to be significant if the *I*^2^ index was > 50%. The possibility of publication bias was examined using the visual inspection of funnel plots and the Egger's regression asymmetry test. To explore and address high heterogeneity, a leave-one-out sensitivity analysis was performed by sequentially removing one study at a time to assess its influence on the pooled effect estimate and identify any potential outliers. All statistical tests were two-sided and used a significance level of *p* < 0.05. All statistical analyses were conducted using R statistical software (Version 4.2.1; R Foundation for Statistical Computing, Vienna, Austria).

## 3. Results

### 3.1. Literature Search and Characteristics of Included Studies

**Figure 1:**
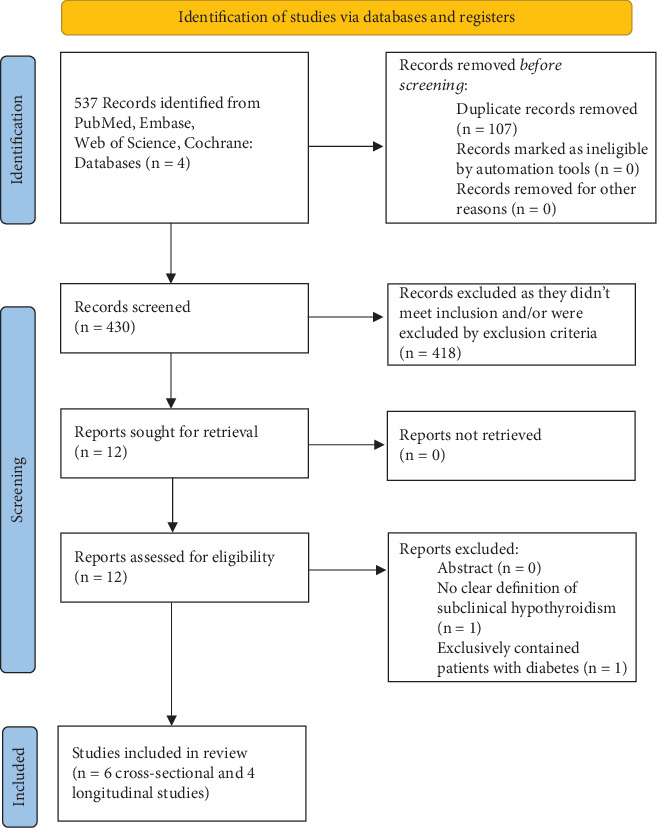
PRISMA diagram.

Figure 1 shows the PRISMA flow diagram of the meta-analysis. After examining the titles and abstracts of these publications and excluding duplicates, we identified 537 potentially eligible studies from five large electronic databases (PubMed, Web of Science, Embase, and Cochrane) from the inception to June, 2025. After comparing those 537 studies with our inclusion and exclusion criteria, we ended up selecting 12 studies to be included in our studies. The 12 included studies were high-quality, full-length, and peer-reviewed. Upon detailed review, we noted that one of the included cross-sectional studies by Tahara et al. [18] presented a discrepancy, reporting an odds ratio (OR) of 4.74 with a confidence interval (CI) of 10.91–12.91, indicating that the OR was below the lower limit of the reported CI so it was excluded, while Sheikhi et al. conducted their study exclusively on patients with diabetes [19]. So, a total of 10 studies ended up being included in our study.

In summary, our meta-analysis pooled 71,332 individuals—31,518 from longitudinal cohorts and 39,814 from cross-sectional studies. The incidence of MASLD in the longitudinal cohorts (mean follow-up 5.7 years) was 12.6%, whereas the cross-sectional prevalence was 30.0%. SCH affected 7.7% of participants in both designs, with balanced sex distributions. The overall mean age was 49.6 years (mean BMI 25.9 kg/m^2^; mean TSH 3.04 mIU/L). Ultrasound was the dominant modality across all studies as mentioned in Table 1.

### 3.2. Cross-Sectional Studies on the Association Between SCH and MASLD

**Figure 2:**
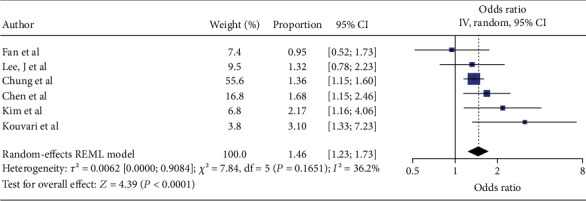
Forest plot of cross-sectional studies showing the relationship between subclinical hypothyroidism and MASLD.

**Figure 3:**
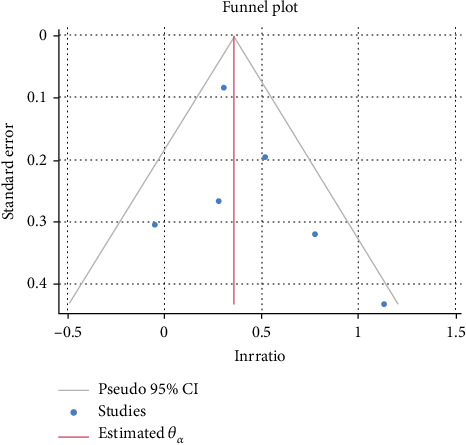
Funnel plot of included cross-sectional studies.

The six cross-sectional studies contributed 39,814 participants. MASLD was identified in 11,953 individuals (30.0%) while SCH was found in 3699 participants (9.3%). Men accounted for 46.4% of the sample. MASLD diagnosis relied on ultrasound in three studies, liver biopsy in two studies, and the Hepatic Steatosis Index (HSI) in one. These six cross-sectional studies collectively suggest that SCH is associated with a higher likelihood of having MASLD. The random-effects meta-analysis yielded a pooled effect of 1.46 (95% CI 1.23–1.73, *p* ≤ 0.001) as illustrated in Figure 2. Importantly, heterogeneity was minimal (*τ*^2^ = 0.006, *I*^2^ = 36.2%, *p* = 0.165), suggesting that the findings were broadly consistent across the included studies. Although some individual studies (e.g., Fan et al. and Lee, J et al.) did not demonstrate statistically significant associations, the overall effect estimate was both robust and precise [25, 26]. The funnel plot shown in Figure 3 appears relatively symmetrical, and the Egger test (*β*_1_ = 0.58, *p* = 0.65) provides no evidence of small-study effects under a random-effects model. A nonparametric trim-and-fill analysis to evaluate potential publication bias revealed minimal influence on our results.

### 3.3. Longitudinal Studies on the Association Between SCH and Incident MASLD

**Figure 4:**
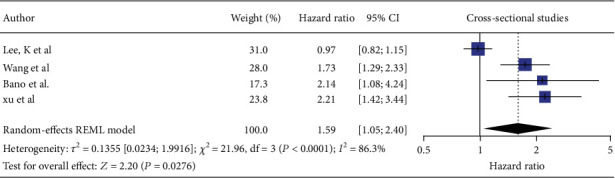
Forest plot of longitudinal studies showing the relationship between MASLD and subclinical hypothyroidism.

**Figure 5:**
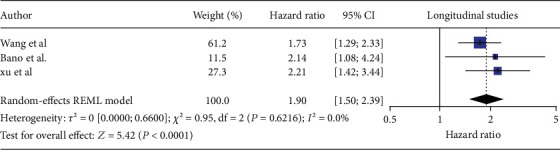
Forrest plot of leave-one-out sensitivity analysis of longitudinal studies showing the relationship between MASLD and subclinical hypothyroidism.

In our meta-analysis of longitudinal studies, the four longitudinal cohorts contributed 31,518 participants. MASLD was identified in 3969 individuals (12.6%) while SCH was found in 2435 participants (7.7%). Men accounted for approximately 47% of participants. MASLD diagnosis relied solely on ultrasound across all four cohorts. In this random-effects meta-analysis examining the association between SCH (exposure) and MASLD incorporating four longitudinal studies (Bano et al., Lee K et al., Wang et al., and Xu et al.) [5, 20–22] initially yielded a modest yet statistically significant pooled hazard ratio of 1.59 (95% CI 1.05–2.40, *p* = 0.017) among patients who have SCH (Figure 4). The results were accompanied by substantial heterogeneity (*I*^2^ = 82.8%, *Q* 3 = 21.8, *p* < 0.001), indicating significant variability among the studies included in the analysis. Meta-regression based on year, continent, and sample size did not result in a significant outcome. Egger's test was not done as only four studies were included in the study. Therefore, a leave-one-out test was done by leaving out Lee et al. [21]. As it was done retrospectively, it not only increased the pooled estimate to 1.90 (95% CI 1.50–2.39) but also eliminated heterogeneity (*I*^2^ = 0%). The distribution of the longitudinal studies by estimate of the association between SCH and the risk of MASLD is plotted in Figures 4 and 5. Applying the GRADE framework, we rated the certainty of evidence for incident MASLD as moderate (Table 2).

## 4. Discussion

This is the first comprehensive meta-analysis specifically investigating the association between SCH and the risk of MASLD. Our analysis included 10 high-quality, peer-reviewed studies involving a large, diverse cohort of 71,332 patients from cross-sectional and longitudinal investigations. Our results demonstrate a significant association between SCH and prevalent MASLD, with a pooled effect of 1.46 times in cross-sectional studies with minimal heterogeneity (*I*^2^ = 36%). Longitudinal studies revealed that SCH was associated with a 59% increased risk of developing incident MASLD (HR 1.59, 95% CI 1.05–2.40; *p* = 0.0276), though with notable heterogeneity (*I*^2^ = 86.3%). While previous meta-analyses by Mantovani et al. examined the broader relationship between primary hypothyroidism and MASLD across approximately 76.5 million individuals with a largely similar OR (1.43, 95% CI 1.23–1.66), our study uniquely focuses on SCH rather than combining it with overt hypothyroidism [4]. This distinction is clinically important, as SCH represents a milder, more prevalent form of thyroid dysfunction that might be overlooked in clinical practice despite its association with liver pathology in our study.

The cross-sectional component of our analysis, comprising five studies and 39,137 individuals, consistently demonstrated an increased likelihood of MASLD in patients with SCH compared to euthyroid individuals. The pooled OR of 1.46 underscores a significant and clinically relevant association. Notably, minimal heterogeneity across studies (*I*^2^ = 36.2%) strengthens confidence in the robustness of this estimate. Diagnostic methodologies varied across the included studies, primarily involving liver ultrasonography (three studies), liver biopsy (one study), and the HSI (one study). Despite these variations, the findings were remarkably consistent, which suggests that the association between SCH and MASLD is not heavily dependent on diagnostic modality. Moreover, the consistent use of biochemical criteria (elevated TSH levels at least above 4 IU with normal free T4) across studies for diagnosing SCH further reinforces the reliability of our outcomes. Interestingly, despite some individual studies (e.g., Fan et al. and Lee et al.) not showing statistically significant associations, the meta-analysis still yielded a significant association [25, 26]. This highlights the importance of aggregating data, as individual studies, particularly smaller ones, may lack sufficient statistical power to identify significant associations independently. Of note, in our analysis, the effect of SCH on MASLD is independent of its effect on dyslipidemia as all the included studies included dyslipidemia as a covariate and the pooled estimate still showed a significant association between MASLD and hypothyroidism.

The longitudinal component of this meta-analysis provides compelling evidence supporting the association between SCH and the increased risk of developing MASLD. Initially, the combined results from four longitudinal studies indicated that individuals with SCH had a modest yet notable higher risk of MASLD by about 59% (HR 1.59, 95% CI 1.05–2.40 *p* = 0.0276). However, the excitement around these findings was somewhat tempered by significant heterogeneity and evidence of publication bias, prompting further scrutiny. Intriguingly, upon performing a sensitivity analysis and excluding the study done retrospectively by Lee et al., the association became even more robust, revealing a 90% increased risk without any residual heterogeneity [21]. The study by Lee et al. might suffer from bias as it did not adjust for diabetes when conducting its analysis which was adjusted for in the other studies and might have blurred the difference between euthyroid patients and those with SCH [21]. When analyzing a prior meta-analysis by Mantovani et al. which combined data from four retrospective longitudinal studies and reported a borderline association (random-effects HR 1.39, 95% CI 0.98–1.97; *I*^2^ = 85%) over a median follow-up of 4.5 years, it is important to note that while their work provided valuable insights, it also faced certain limitations [4]. These included high heterogeneity, the absence of a consistent definition of SCH in the methodology and the combination of primary hypothyroidism and SCH together. Since our meta-analysis focused on identifying the relationship between SCH and MASLD, our study was more thorough and targeted to SCH in selecting studies that explicitly identified MASLD as an outcome, which we believe strengthened the robustness of our findings.

It may be argued that the lower prevalence of MASLD observed in our study, compared with the global estimate of 38%, reflects differences in study design and population characteristics rather than solely selection bias [29]. The retrospective longitudinal cohort demonstrated a prevalence of 12.6%, likely influenced by inclusion of participants with available follow-up data, which may underestimate true prevalence due to incomplete capture or loss of higher-risk individuals. In contrast, the cross-sectional cohort showed a prevalence of 30%, more consistent with population-based estimates. These findings highlight how retrospective designs may underestimate disease burden and emphasize the importance of standardized, prospective data collection to better characterize MASLD prevalence and progression.

Building on these epidemiologic considerations, a recent article from EMJ Hepatology (2025), “Primary MASLD” and “Secondary MASLD” represent distinct entities with differing pathophysiological mechanisms, clinical outcomes, and therapeutic strategies [30]. While hypothyroidism is recognized as a potential cause of Secondary MASLD, the guideline does not explicitly delineate the categorization of patients with SCH [30]. The observed association between SCH and MASLD in our analysis underscores the need for future outcome-oriented studies to elucidate whether MASLD secondary to SCH aligns more closely with the metabolic features of primary MASLD or the etiological framework of secondary MASLD. Furthermore, it remains to be determined whether these cases respond to levothyroxine therapy or require management approaches similar to those used for primary MASLD; preliminary evidence indicates potential benefit [28].

Translating these findings into clinical practice, when comparing the risk of MASLD in patients with SCH to one of the traditional metabolic risk factors, we observed that SCH conferred a 1.90-fold increase in odds, whereas hypertension was associated with a 1.66-fold increase (95% CI 1.38–2.01) [31]. Given this growing body of evidence, clinicians should incorporate MASLD screening into the management of patients diagnosed with SCH particularly if they also have another metabolic risk factor such as diabetes, dyslipidemia, obesity, or hypertension. Conversely, individuals identified with MASLD should undergo routine thyroid function testing to detect SCH early and guide comprehensive risk reduction.

## 5. Limitations

This meta-analysis has a few limitations inherent to the designs of the included studies. First, the retrospective nature of cross-sectional studies prevents establishing a clear timeline between the development of primary hypothyroidism and MASLD. Second, although most studies adjusted for known risk factors and potential confounders, the possibility of residual confounding by unmeasured factors cannot be completely ruled out. Third, a key limitation of this study is the lack of sex-specific analyses, despite evidence of marked sexual dimorphism in MASLD pathophysiology [28, 32, 33]. In light of this, understanding sex-related differences could provide important insights into disease mechanisms and outcomes. However, we were unable to assess the influence of sex on the association between SCH and MASLD, as the included studies did not report sex-stratified data. Future studies should address this gap to clarify potential sex-based variations. Finally, while a selective reporting bias cannot be entirely excluded, our comprehensive search strategy makes it unlikely that we missed any published studies.

## 6. Conclusion

In this comprehensive meta-analysis of both cross-sectional and longitudinal studies, SCH was consistently associated with an increased prevalence of MASLD, as well as a heightened risk of developing MASLD over time. The pooled data indicate that individuals with SCH have approximately 1.4-fold greater odds of having MASLD in cross-sectional investigations and up to a 1.65-fold higher hazard of incident MASLD in longitudinal cohorts—findings that remained robust in sensitivity analyses despite some heterogeneity. These results underscore the importance of considering routine screening for MASLD in patients with SCH. Future well-designed, prospective trials are warranted to clarify whether therapeutic interventions for SCH can mitigate MASLD onset or progression.

## Figures and Tables

**Table 1:** Summary of included studies.

**Longitudinal studies**															
**First author, Ref.**	**Year**	**Country**	**Sample size (** **n** **)**	**Followup (yr)**	**Age (yr)**	**BMI (kg/m** ^**2**^ **)**	**% male**	**TSH (mIU/L)**	**MASLD diagnosis modality**	**MASLD patients (** **n** **)**	**SCH threshold (mIU/L)**	**SCH patients (** **n** **)**	**Covariates**	**HR (95% CI)**	**NOS**

Bano et al. [20]	2016	Netherlands	9419	10	68	27.2	44	1.9	US	1.217	4	408	1.2	2.14 (1.04–4.07)	9
Lee, K et al. [21]	2015	South Korea	18,544	4	38	22.5	53	2.3	US	2348	4.2	1301	1.2	0.97 (0.81–1.14)	8
Wang et al. [5]	2024	China	2,901	1.3	50	23.5	44	2.8	US	306	4.5	399	1.2	1.73 (1.29–2.33)	9
Xu et al. [22]	2012	China	654	4.92	N/A	N/A	N/A	N/A	US	98	4.5	327	1.2	2.21 (1.42–3.44)	8

**Cross-sectional studies**															
**First author, Ref.**	**Year**	**Country**	**Sample size (** **n** **)**	**Age (yr)**		**BMI (kg/m** ^**2**^ **)**	**% male**	**TSH (mIU/L)**	**MASLD diagnosis modality**	**MASLD patients (** **n** **)**	**SCH threshold (mIU/L)**	**SCH patients (** **n** **)**	**Covariates**	**OR (95% CI)**	**NOS**

Chung et al. [23]	2012	South Korea	4648	48.6		22.9	37.6	5.09	US	875	4.1	2189	1.2	1.36 (1.16–1.61)	8
Kim et al. [24]	2018	South Korea	425	53		27.8	52	3.4	Biopsy	425	4.5	59	1.2	2.17 (1.16–4.07)	8
Lee, J et al. [25]	2018	South Korea	3452	44.5		NA	44.8	NA	HSI	1161	6.68	128	1.2	1.32 (0.78–2.22)	8
Fan et al. [26]	2023	China	19946	47.31		23.5	52.78		US	5495	4.5	232	1.2	0.95 (0.51–1.68)	8
Chen et al. [27]	2023	China	10666	41.7		26.4	51.45	1.88	US	3320	4.5	414	1.2	1.68 (1.16–2.43)	9
Kouvari et al. [28]	2024	USA	677	51.2		35.2	40	NA	Biopsy	677	4	57	2	3.1 (1.33–7.23)	9

*Note:* Covariate adjustment: (1) age, sex, and BMI/waist circumference; (2) diabetes, hypertension/cardiovascular disease, and dyslipidemia.

Abbreviations: HR, hazard ratio; MASLD, metabolic dysfunction–associated steatotic liver disease; NOS, Newcastle–Ottawa scale (quality assessment tool for non-randomized studies); OR, odds ratio; PRISMA, Preferred Reporting Items for Systematic Reviews and Meta-Analyses; SCH, subclinical hypothyroidism; TSH, thyroid-stimulating hormone.

**Table 2:** GRADE summary of findings.

**Outcome**	**Effect estimate (95% CI)**	**Certainty**	**Comments**
Incidence of MASLD in subclinical hypothyroidism	HR 1.9 (1.5–2.39)^a^	Moderate	Downgraded one level because the evidence comes from observational cohorts

^a^Prospective-only sensitivity analysis; removes heterogeneity that was driven by one retrospective cohort.

## Nomenclature

MASLD: metabolic dysfunction–associated steatotic liver disease
PRISMA: Preferred Reporting Items for Systematic Reviews and Meta-Analyses
SCH: subclinical hypothyroidism
TSH: thyroid-stimulating hormone
OR: odds ratio
HR: hazard ratio
NOS: Newcastle–Ottawa Scale (for assessing quality of nonrandomized studies)

## Supporting Information

Supporting Information 1 Material S1: PRISMA 2020 checklist: This checklist outlines the reporting items recommended in the PRISMA 2020 statement. It specifies where each item is addressed in the manuscript to ensure transparency, reproducibility, and adherence to systematic review reporting standards.

Supporting Information 2 Table S1: Search strategy: This table provides the full electronic search strategy, including all keywords, subject headings, and Boolean operators, to enhance reproducibility of the systematic review.
