# Supplementary material for: Association Between Subclinical Hypothyroidism and MASLD: A Systematic Review and Meta-Analysis
Source: Int J Hepatol. 2025 Nov 29;2025:8133686. doi: 10.1155/ijh/8133686 (PMC12681400; doi:10.1155/ijh/8133686)
Supplement: Supporting Information 2 — Table S1: Search strategy: This table provides the full electronic search strategy, including all keywords, subject headings, and Boolean operators, to enhance reproducibility of the systematic review. [file 8133686.f2.docx]

**Supplemental Table 1:** search Strategy
*This table provides the full electronic search strategy, including all keywords, subject headings, and Boolean operators, to enhance reproducibility of the systematic review.*

| Database | Search query |
| --- | --- |
| Pubmed | (("Hypothyroidism"[Mesh] AND subclinical[tiab]) OR "subclinical hypothyroidism"[tiab]) AND ( "Non-alcoholic Fatty Liver Disease"[Mesh] OR "non-alcoholic fatty liver disease"[tiab] OR NAFLD[tiab] OR "non-alcoholic steatohepatitis"[tiab] OR NASH[tiab] OR "metabolic dysfunction-associated steatotic liver disease"[tiab] OR MASLD[tiab] OR "metabolic dysfunction-associated steatotic hepatitis"[tiab] OR "metabolic dysfunction-associated fatty liver disease"[tiab] OR MAFLD[tiab] ) |
| Web of Science | (TS=("subclinical hypothyroidism") AND TS=("non-alcoholic fatty liver disease" OR "NAFLD" OR "non-alcoholic steatohepatitis" OR "NASH" OR "metabolic dysfunction-associated steatotic liver disease" OR "MASLD" OR "metabolic dysfunction-associated steatotic hepatitis" OR "MASH" OR "metabolic dysfunction-associated fatty liver disease" OR "MAFLD") ) |
| Embase | ('Non-alcoholic Fatty Liver Disease':ti,ab,kw OR 'NAFLD':ti,ab,kw OR 'Metabolic dysfunction-associated steatotic liver disease':ti,ab,kw OR 'MASLD':ti,ab,kw OR 'Metabolic dysfunction-associated steatotic hepatitis':ti,ab,kw OR 'MASH':ti,ab,kw) AND ('Subclinical Hypothyroidism':ti,ab,kw OR 'mild thyroid failure':ti,ab,kw) {No Related Terms} |
| Cochrane | "subclinical hypothyroidism" AND ("non-alcoholic fatty liver disease" OR NAFLD OR "non-alcoholic steatohepatitis" OR NASH OR "metabolic dysfunction-associated steatotic liver disease" OR MASLD OR "metabolic dysfunction-associated steatotic hepatitis" OR MASH OR "metabolic dysfunction-associated fatty liver disease" OR MAFLD) |
| Clinicaltrials.gov | "subclinical hypothyroidism" AND ("non-alcoholic fatty liver disease" OR NAFLD OR "non-alcoholic steatohepatitis" OR NASH OR "metabolic dysfunction-associated steatotic liver disease" OR MASLD OR "metabolic dysfunction-associated steatotic hepatitis" OR MASH OR "metabolic dysfunction-associated fatty liver disease" OR MAFLD) |
